# Plasma SPARC Concentrations in Patients with Subsequent Shunt-Dependent Hydrocephalus After Aneurysmal Subarachnoid Hemorrhage

**DOI:** 10.3390/ijms27219929

**Published:** 2026-11-06

**Authors:** Koichi Hakozaki, Hideki Nakajima, Yotaro Kitano, Hirofumi Nishikawa, Fumihiro Kawakita, Ryuta Yasuda, Hidenori Suzuki, pSEED Group

**Affiliations:** Department of Neurosurgery, Graduate School of Medicine, Mie University, 2-174 Edobashi, Tsu 514-8507, Japan; hakozakik@med.mie-u.ac.jp (K.H.); nakajimahideki0131@med.mie-u.ac.jp (H.N.); ykitano@med.mie-u.ac.jp (Y.K.); hnishikawa0220@med.mie-u.ac.jp (H.N.); fumiwo0219@med.mie-u.ac.jp (F.K.); ryasuda@med.mie-u.ac.jp (R.Y.)

**Keywords:** shunt-dependent hydrocephalus, matricellular protein, secreted protein acidic and rich in cysteine, subarachnoid hemorrhage

## Abstract

Shunt-dependent hydrocephalus (SDHC) is a delayed complication of aneurysmal subarachnoid hemorrhage (SAH), and meningeal fibrotic remodeling may contribute to its development. Secreted protein acidic and rich in cysteine (SPARC) regulates collagen processing and extracellular matrix organization. We examined whether serial plasma SPARC concentrations were associated with subsequent SDHC development. We retrospectively analyzed prospectively collected data from 186 patients with aneurysmal SAH enrolled in a multicenter registry. Plasma SPARC was measured at days 1–3, 4–6, 7–9, and 10–12 and analyzed using log-scale linear mixed-effects models. SDHC developed in 54 patients (29.0%). Model-estimated SPARC concentrations increased across periods in both SDHC and no-SDHC groups without a significant SDHC-by-period interaction, whereas all the SDHC/no-SDHC geometric mean ratios were below 1. The clearest difference occurred at days 4–6 and remained significant after adjustment for age, admission World Federation of Neurological Surgeons grade V, modified Fisher grade 4, and acute hydrocephalus (geometric mean ratio, 0.73; 95% confidence interval, 0.57–0.93; Holm-adjusted *p* = 0.040). In a post hoc exploratory multivariable analysis, each 10 ng/mL increase in SPARC at days 4–6 was associated with lower odds of subsequent SDHC development (adjusted odds ratio, 0.91; 95% confidence interval, 0.84–0.98; *p* = 0.011). Lower plasma SPARC concentrations, particularly at days 4–6, were associated with subsequent SDHC development.

## 1. Introduction

Aneurysmal subarachnoid hemorrhage (SAH) remains a severe cerebrovascular disease associated with substantial morbidity and mortality [1]. Shunt-dependent hydrocephalus (SDHC) is a delayed complication that develops in approximately one in five patients after aneurysmal SAH, requires permanent cerebrospinal fluid (CSF) shunting, and is associated with poor functional outcomes [2]. Although CSF shunting is an established treatment, shunt malfunction, obstruction, and infection remain important clinical problems [3]; in a long-term cohort of patients shunted after aneurysmal SAH, 32% underwent shunt revision [4]. A better understanding of the pathophysiology of SDHC might help resolve these issues, facilitating the development of preventive or non-shunt therapeutic strategies.

Blood and its degradation products in the subarachnoid space may impair CSF circulation and clearance through mechanical obstruction and inflammatory responses [5]. Fibrosis of the leptomeninges and arachnoid granulations has long been implicated in the persistent impairment of CSF absorption after SAH [5,6]. Experimental and clinical findings have expanded this conventional concept [7,8,9]. In a mouse SAH model, an expansion of meningeal fibroblasts and increased collagen biosynthesis-related transcriptional activity around meningeal lymphatic vessels were observed at 72 h, when meningeal lymphatic drainage had begun to recover [10]. In a separate non-SAH model, excessive peri-lymphatic collagen deposition impaired meningeal lymphatic drainage [11]. These findings raise the possibility that fibroblast-mediated remodeling initially supports tissue repair, whereas excessive or persistent collagen deposition may impair CSF clearance through meningeal pathways. However, how meningeal fibrosis and collagen deposition develop in humans after SAH and contribute to SDHC remains poorly understood.

Secreted protein acidic and rich in cysteine (SPARC) is a collagen-binding matricellular glycoprotein. SPARC and its derived peptides can exert both pro- and anti-angiogenic effects [12,13], while SPARC has also shown pro- and anti-inflammatory effects in experimental models [14,15]. In extracellular matrix remodeling, SPARC regulates procollagen I processing, incorporation into the extracellular matrix, and collagen fibrillogenesis [16,17]. Experimental studies have also implicated SPARC in fibrotic remodeling [18,19]; SPARC silencing reduced type I collagen expression and attenuated bleomycin-induced skin and lung fibrosis in mice [19]. SPARC expression is upregulated in cerebral endothelial cells after experimental SAH, and plasma SPARC concentrations increase after aneurysmal SAH in humans [20,21,22]. However, whether serial plasma SPARC concentrations are associated with subsequent SDHC development remains unknown. Clarifying this relationship may help us better understand the pathophysiology of SDHC, including meningeal remodeling and fibrosis after aneurysmal SAH.

In the present study, we examined serial plasma SPARC concentrations during the first 12 days after aneurysmal SAH and their association with subsequent SDHC development. We hypothesized that serial plasma SPARC concentrations would differ between patients who did and did not develop SDHC.

## 2. Results

### 2.1. Patient Characteristics

**Figure 1:**
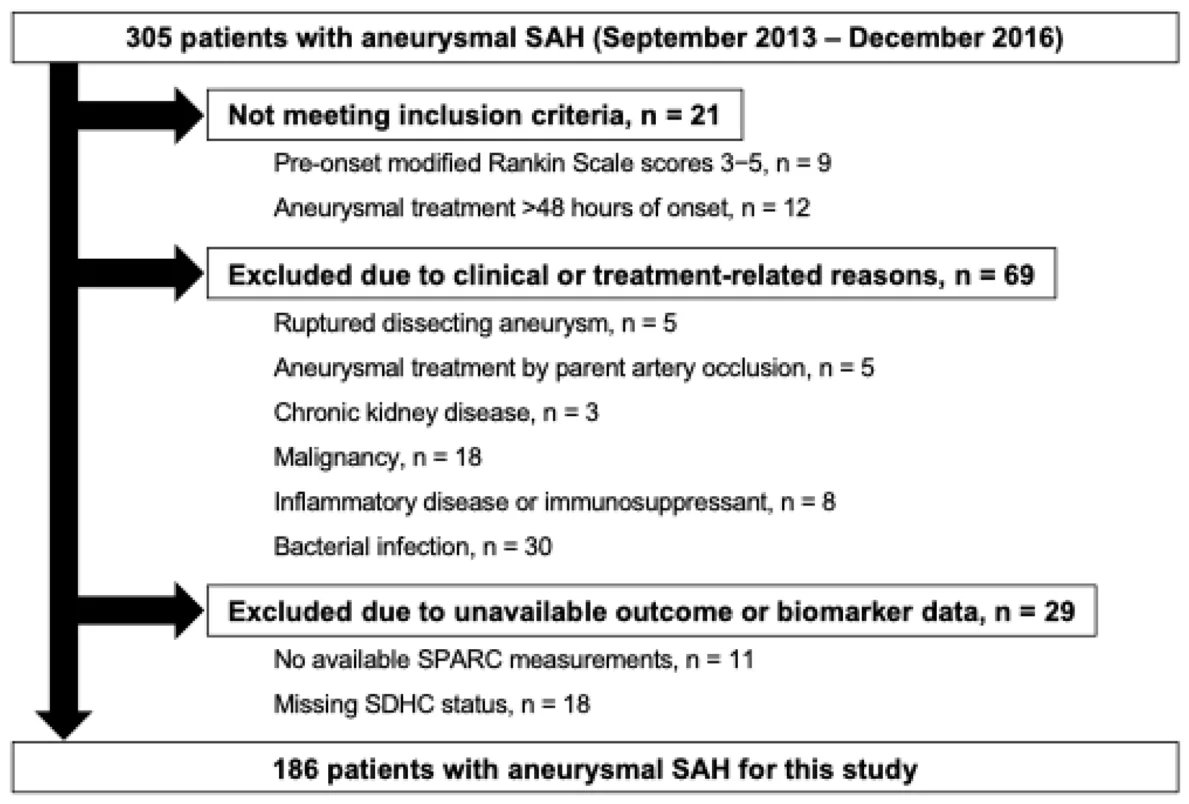
A flow chart showing the included and excluded patients in this study. SAH, subarachnoid hemorrhage; SDHC, shunt-dependent hydrocephalus; SPARC, secreted protein acidic and rich in cysteine.

During the study period, 305 consecutive patients with SAH were registered in the Prospective Registry for Searching Mediators of Neurovascular Events After Aneurysmal SAH (pSEED). A flow chart of the participant selection is shown in Figure 1. After applying the eligibility criteria, 186 patients with aneurysmal SAH were included in the main analysis. The median age of the analysis cohort was 65.5 years (interquartile range, 50.2–73.0 years), and 134 patients (72.0%) were female. A pre-onset modified Rankin Scale (mRS) score of 0 was recorded in 172 patients (92.5%). Admission World Federation of Neurological Surgeons (WFNS) grade V and modified Fisher grade 4 were observed in 39 (21.0%) and 64 (34.4%) patients, respectively. Acute hydrocephalus was present in 63 patients (33.9%), and 17 ruptured aneurysms (9.1%) were located in the posterior circulation. SDHC developed in 54 patients (29.0%) (Table 1). SPARC measurements were available at all four sampling periods in 184 patients. Two patients lacked measurements for days 4–6 because of insufficient sample volumes but had measurements for the other three periods, yielding 742 observations for the longitudinal analysis. Ninety-day mRS scores were unavailable in two other patients, who were retained because their SDHC status was known.

With the exception of 30 cases of bacterial infection identified during hospitalization, all other patients were excluded either because the underlying condition was already present at the time of admission or aneurysmal obliteration, or because SPARC measurements or SDHC status were unknown. Among the 119 excluded cases, both SPARC measurements and SDHC status were available for 74 cases, 29 of which were excluded due to a bacterial infection. Therefore, a separate analysis was conducted for the 29 cases in which a bacterial infection was confirmed during hospitalization.

### 2.2. Clinical Variables According to SDHC Status

Clinical variables according to SDHC status are summarized in Table 1. Patients who developed SDHC were older than those who did not (median, 72.0 vs. 60.0 years; absolute standardized difference [ASD], 0.802). Admission WFNS grade and modified Fisher grade also showed relatively large between-group differences (ASD, 0.719 and 0.587, respectively), with higher proportions of admission WFNS grade V (37.0% vs. 14.4%) and modified Fisher grade 4 (50.0% vs. 28.0%) in the SDHC group. Acute hydrocephalus was more frequent in the SDHC group (63.0% vs. 22.0%; ASD, 0.911). CSF drainage was also more frequently performed in the SDHC group (63.0% vs. 23.5%; ASD, 0.869), particularly ventricular drainage (57.4% vs. 12.1%; ASD, 1.081). Post-SAH clinical events, including delayed cerebral ischemia (DCI) (27.8% vs. 11.4%; ASD, 0.423), angiographic vasospasm (AVSP) (48.1% vs. 26.5%; ASD, 0.459), and delayed cerebral infarction (DCIn) (33.3% vs. 19.7%; ASD, 0.313), were more frequent in the SDHC group. At 90 days, an mRS score of 0–2 was recorded in 14 patients (26.4%) with SDHC and 102 patients (77.9%) without SDHC (ASD, 1.201).

### 2.3. Temporal Profiles of Plasma SPARC Concentrations According to SDHC Status

**Figure 2:**
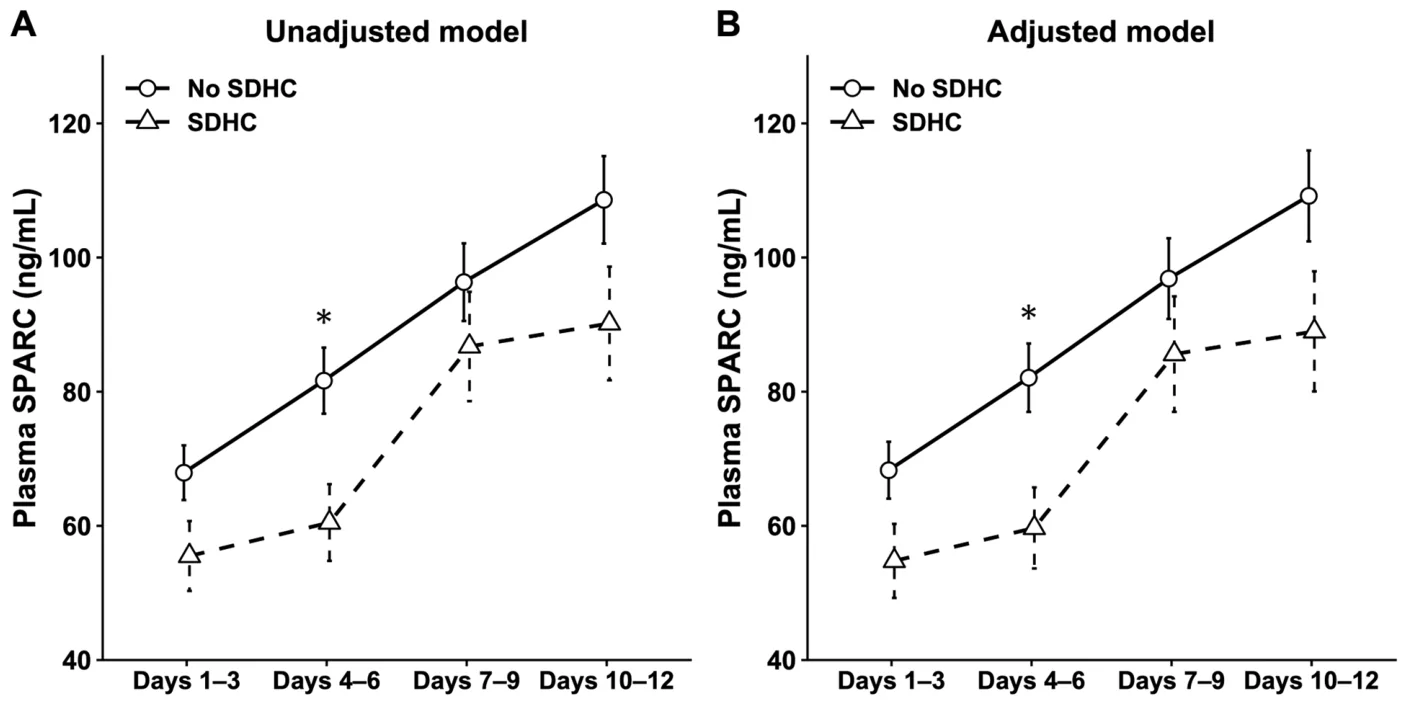
Temporal changes in plasma secreted protein acidic and rich in cysteine (SPARC) concentrations according to subsequent development of shunt-dependent hydrocephalus (SDHC) after aneurysmal subarachnoid hemorrhage. Model-estimated plasma SPARC concentrations are shown for patients who did and did not subsequently develop SDHC. Numbers of patients with available SPARC measurements for each sampling period are provided in Supplementary Table S1. (**A**) Unadjusted model. (**B**) Model adjusted for age, admission World Federation of Neurological Surgeons grade V, modified Fisher grade 4, and acute hydrocephalus. Both models include SDHC status, sampling period, and their interaction as fixed effects and a patient-specific random intercept. Plasma SPARC concentrations were log-transformed before analysis. Back-transformed estimated marginal means are presented as geometric means, and error bars indicate standard errors. * Holm-adjusted *p* < 0.05 for the between-group contrast within the corresponding sampling period.

Model-estimated plasma SPARC concentrations increased across the four sampling periods in both groups (Figure 2 and Supplementary Table S1). The SDHC-by-period interaction was not significant in either the unadjusted model or the model adjusted for age, admission WFNS grade V, modified Fisher grade 4, and acute hydrocephalus (P for interaction = 0.323 for both), providing no evidence that the temporal profiles differed between the groups. However, the point estimates of the geometric mean ratios (GMRs) were below 1 at all four sampling periods, indicating lower model-estimated plasma SPARC concentrations in the SDHC group. In the unadjusted model, the clearest between-group difference was observed at days 4–6 (GMR, 0.74; 95% confidence interval [CI], 0.59–0.92; Holm-adjusted *p* = 0.031). The corresponding estimate in the adjusted model was similar (GMR, 0.73; 95% CI, 0.57–0.93; Holm-adjusted *p* = 0.040). No between-group differences remained significant after Holm adjustment at the other sampling periods (Supplementary Table S2). In the sensitivity analysis excluding six patients with a 90-day mRS score of 6 and no recorded SDHC, the between-group difference at days 4–6 remained significant in the unadjusted model (GMR, 0.73; 95% CI, 0.58–0.91; Holm-adjusted *p* = 0.021). The corresponding estimate in the adjusted model was similar (GMR, 0.70; 95% CI, 0.55–0.90; Holm-adjusted *p* = 0.023) (Supplementary Figure S1 and Table S3).

### 2.4. Exploratory Subgroup Analyses of Plasma SPARC Concentrations by SDHC Status

**Figure 3:**
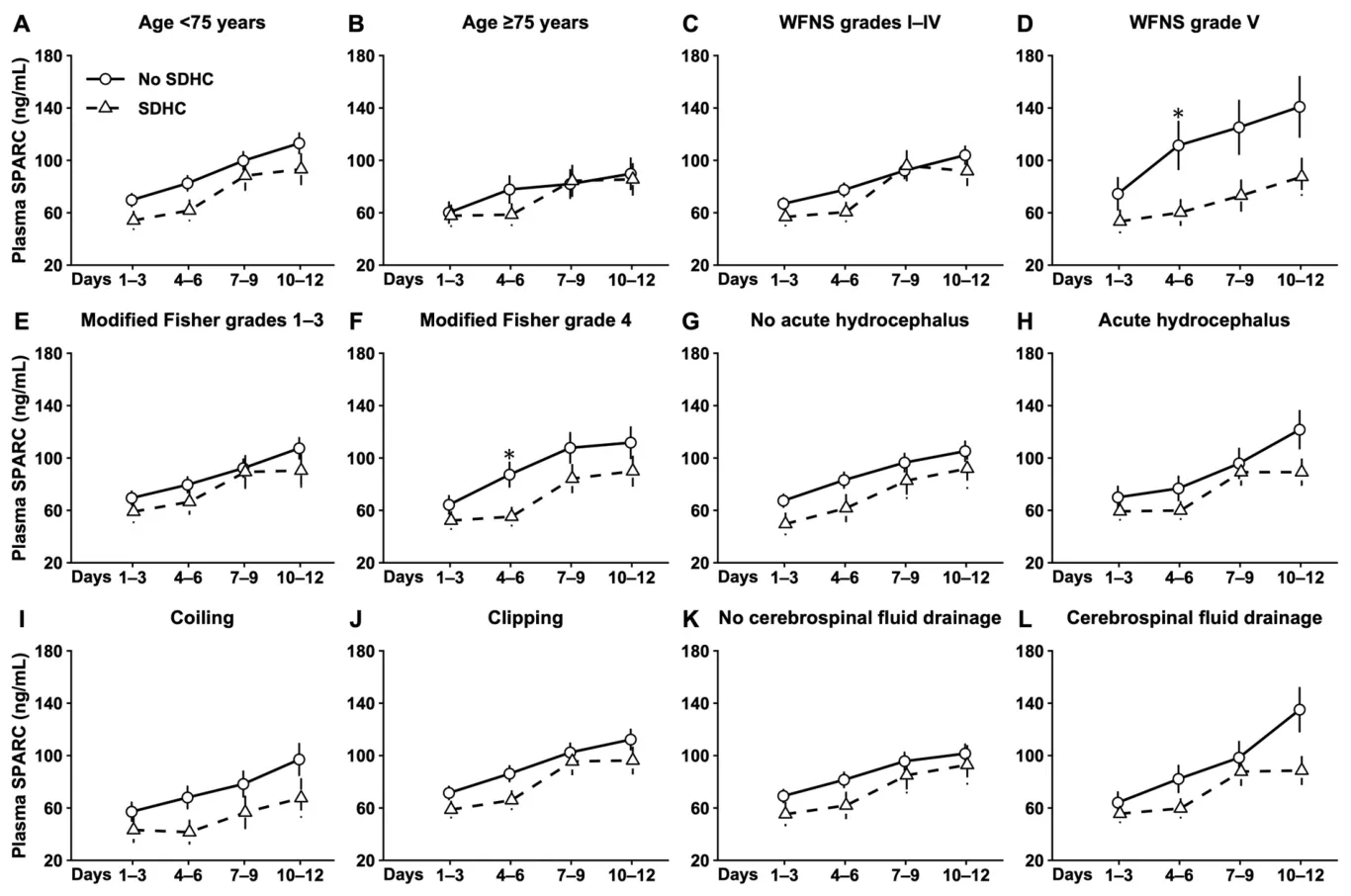
Exploratory subgroup analyses of temporal changes in plasma secreted protein acidic and rich in cysteine (SPARC) concentrations according to subsequent development of shunt-dependent hydrocephalus (SDHC). Model-estimated plasma SPARC concentrations are shown for patients who did and did not subsequently develop SDHC, stratified by age < 75 years (**A**), age ≥ 75 years (**B**), admission World Federation of Neurological Surgeons (WFNS) grades I–IV (**C**), admission WFNS grade V (**D**), modified Fisher grades 1–3 (**E**), modified Fisher grade 4 (**F**), absence of acute hydrocephalus (**G**), presence of acute hydrocephalus (**H**), coiling (**I**), clipping (**J**), absence of cerebrospinal fluid drainage (**K**), and presence of cerebrospinal fluid drainage (**L**). Numbers of patients with available SPARC measurements by SDHC status, sampling period, and subgroup are provided in Supplementary Table S4. Each panel is based on a separate unadjusted linear mixed-effects model that includes SDHC status, sampling period, and their interaction as fixed effects and a patient-specific random intercept. Plasma SPARC concentrations were log-transformed before analysis. Back-transformed estimated marginal means are presented as geometric means, and error bars indicate standard errors. * Holm-adjusted *p* < 0.05 for the between-group contrast within the corresponding sampling period. Holm adjustment is applied across the four period-specific contrasts within each subgroup.

Exploratory subgroup analyses stratified by age, admission WFNS grade, modified Fisher grade, acute hydrocephalus, treatment modality, and CSF drainage are shown in Figure 3 and Supplementary Table S4. No SDHC-by-period interaction was significant in any subgroup. However, in most subgroup strata, the GMR point estimates were below 1 at all four sampling periods, indicating generally lower model-estimated plasma SPARC concentrations in patients with SDHC. At days 4–6, GMRs were below 1 in all subgroup strata, and the between-group difference remained significant after Holm adjustment in patients with admission WFNS grade V (GMR, 0.54; 95% CI, 0.34–0.85; Holm-adjusted *p* = 0.034) and modified Fisher grade 4 (GMR, 0.63; 95% CI, 0.46–0.87; Holm-adjusted *p* = 0.023).

### 2.5. Multivariable Logistic Regression Analysis of the Association Between Plasma SPARC Concentration at Days 4–6 and Subsequent SDHC

To further estimate the magnitude of the association between the plasma SPARC concentration at days 4–6 and subsequent development of SDHC, multivariable logistic regression was performed as a post hoc exploratory analysis. After adjustment for age, admission WFNS grade V, modified Fisher grade 4, and acute hydrocephalus, each 10 ng/mL increase in plasma SPARC concentration at days 4–6 was associated with lower odds of subsequent SDHC development (adjusted odds ratio [aOR], 0.91; 95% CI, 0.84–0.98; *p* = 0.011). Acute hydrocephalus (aOR, 3.74; 95% CI, 1.71–8.17; *p* < 0.001), admission WFNS grade V (aOR, 2.84; 95% CI, 1.14–7.11; *p* = 0.026), and older age (aOR per 10-year increase, 1.47; 95% CI, 1.11–1.95; *p* = 0.008) were associated with higher odds of SDHC. Modified Fisher grade 4 was not clearly associated with SDHC in this model (aOR, 1.18; 95% CI, 0.52–2.68; *p* = 0.691) (Table 2).

After additional adjustment for CSF drainage, higher plasma SPARC concentrations at days 4–6 remained associated with lower odds of SDHC (aOR per 10 ng/mL increase, 0.90; 95% CI, 0.84–0.98; *p* = 0.012) (Supplementary Table S5).

In the exploratory model comparison, adding plasma SPARC at days 4–6 increased the area under the receiver operating characteristic (ROC) curve (AUC) in the model-development data from 0.796 to 0.821. The corresponding bootstrap optimism-corrected AUCs were 0.781 and 0.803, respectively. The continuous net reclassification improvement (NRI) was 0.267 (95% CI, −0.040–0.574), and the integrated discrimination improvement (IDI) was 0.037 (95% CI, 0.010–0.063). ROC curves and detailed estimates with 95% CIs are presented in Supplementary Figure S2 and Table S6, respectively.

### 2.6. Additional Analyses of Patients with Bacterial Infections

Among the 30 patients excluded primarily due to bacterial infections, 29 had available SPARC measurements and known SDHC status. These patients were older and more frequently had admission WFNS grade V and modified Fisher grade 4 than those in the main analysis cohort (Supplementary Table S7). Including these 29 patients yielded 215 patients and 858 measurements. After adjustment for the four clinical covariates and bacterial infection, the between-group difference at days 4–6 was no longer significant after Holm adjustment (GMR, 0.81; 95% CI, 0.65–1.00; Holm-adjusted *p* = 0.214) (Supplementary Figure S3 and Table S8). Among patients with infections, no period-specific contrast was significant after Holm adjustment in either the central nervous system (CNS; n = 10; 9 cases of meningitis and 1 of brain abscess) or non-CNS (n = 19; 17 cases of pneumonia, 1 of puncture-site infection and 1 of thigh abscess) stratum. At days 4–6, the unadjusted GMR was 1.00 (95% CI, 0.45–2.24; Holm-adjusted *p* = 1.000) in the CNS stratum and 1.63 (95% CI, 0.79–3.37; Holm-adjusted *p* = 0.738) in the non-CNS stratum (Supplementary Figure S4 and Table S9). However, in the logistic regression analysis of 213 patients at days 4–6, including 65 with SDHC, higher plasma SPARC concentrations remained associated with lower odds of SDHC after adjustment for the four clinical covariates and bacterial infection (aOR per 10 ng/mL increase, 0.94; 95% CI, 0.88–1.00; *p* = 0.038) (Supplementary Table S10).

## 3. Discussion

The principal finding of this study was that patients who subsequently developed SDHC showed lower model-estimated plasma SPARC concentrations at all four sampling periods. The clearest between-group difference was observed at days 4–6 after aneurysmal SAH. The SDHC-by-period interaction was not significant, and plasma SPARC concentrations increased across the four sampling periods in both groups. The between-group difference at days 4–6 persisted after adjustment for relevant clinical factors. Consistent with this finding, a post hoc exploratory multivariable logistic regression analysis showed that higher plasma SPARC concentrations at days 4–6 were associated with lower odds of subsequently developing SDHC after adjustment for the same factors. These findings suggest that lower circulating SPARC concentrations are associated with subsequent SDHC development after aneurysmal SAH.

The observed association appeared to reflect a generally maintained difference in concentration levels, with plasma SPARC concentrations already tending to be lower in the SDHC group at the beginning of the observation period, rather than an abrupt change specific to that group. Although the nonsignificant interaction does not establish that the temporal profiles were identical, it provides no evidence of clearly different patterns between the groups. In the exploratory subgroup analyses, the GMRs were below 1 across all four sampling periods in most strata and were below 1 in every stratum at days 4–6. The association between plasma SPARC concentrations at days 4–6 and SDHC persisted after additional adjustment for CSF drainage, but the biological processes underlying this association remain unclear.

Although the patients excluded due to bacterial infections were older and more often had severe SAH, the GMRs at days 4–6 were below 1 across both age strata and the severity strata in the main analysis cohort (Figure 3). The days 4–6 between-group difference was attenuated after including infection cases. In the infection-site analyses at days 4–6, the point estimate suggested little difference by SDHC status in the CNS stratum, whereas the non-CNS stratum showed a point estimate in the opposite direction to the main analysis, with higher SPARC concentrations in patients with SDHC (Supplementary Figure S4 and Table S9). These findings suggest that a bacterial infection may have a greater impact on plasma SPARC concentrations than the SDHC status itself. Furthermore, the magnitude of this impact might vary depending on whether the infection involves the CNS and on the severity of the infection. However, given the imprecise estimates and lack of information regarding infection severity, this interpretation remains speculative. Nevertheless, in the expanded cohort, higher plasma SPARC concentrations at days 4–6 remained associated with lower odds of SDHC in the logistic regression model adjusted for clinical covariates and bacterial infection.

### 3.1. The Transition Period from the Acute Inflammatory Response Following SAH to Meningeal Remodeling

To interpret why the between-group difference was most apparent at days 4–6, it is necessary to consider the transition from acute inflammatory responses to meningeal tissue remodeling after SAH. In a prospective study of patients with SAH, inflammatory and coagulation-related factors in early CSF samples were associated with subsequent chronic hydrocephalus [23]. In a mouse SAH model, myeloid cell infiltration into the meninges and impaired meningeal lymphatic drainage at 24 h were followed at 72 h by attenuation of the inflammatory response, a marked expansion of meningeal fibroblasts, and increased collagen biosynthesis-related transcriptional activity around meningeal lymphatic vessels [10]. Because this fibroblast response may contribute to tissue repair, it does not necessarily represent pathological fibrosis at that stage, although it may be related to subsequent fibrotic remodeling.

In analyses of aged dura and a mouse model with constitutively active transforming growth factor beta (TGF-β) receptor 1 signaling in dural fibroblasts, peri-lymphatic type I collagen accumulation impaired meningeal lymphatic drainage; complementary experiments showed that increased matrix stiffness disrupted lymphatic endothelial junctional integrity and lymphangiogenesis, thereby compromising CSF clearance [11]. Furthermore, in a rat SAH model, inhibition of the TGF-β1/Smad/connective tissue growth factor pathway by decorin attenuated both extracellular matrix accumulation, including type I collagen, and chronic hydrocephalus [24]. Taken together, these findings support a model in which the fibroblast and extracellular matrix responses after SAH may initially contribute to tissue repair but, if excessive or persistent, may impair CSF clearance through collagen deposition and tissue stiffening. The timing of the clearest SPARC difference at days 4–6 may therefore be consistent with the transition from acute inflammation to fibroblast-mediated meningeal remodeling, although this interpretation remains speculative.

### 3.2. Functional Differences Between Other Matricellular Proteins and SPARC in the Pathology of SDHC

Based on this fibrosis-related framework, we initially expected that patients who developed SDHC would have enhanced local SPARC-mediated extracellular matrix responses and that part of this increase would be reflected in the circulation, resulting in higher plasma SPARC concentrations. This expectation was consistent with previous studies. Plasma and CSF levels of osteopontin (OPN) followed a similar temporal course after aneurysmal SAH [25]. In addition, plasma OPN, thrombin-cleaved OPN N-half, and fibulin-5 (FBLN5) were elevated in patients who subsequently developed SDHC or chronic hydrocephalus [26,27,28]. However, although these proteins are all associated with fibrosis, their mechanisms of action and behavior within local tissues differ.

OPN primarily regulates integrin-mediated cellular signaling involved in inflammation, wound healing, and fibroblast responses. In OPN N-half, thrombin cleavage exposes the SVVYGLR sequence, enhancing fibroblast migration, Smad signaling, α-smooth muscle actin expression, and type III collagen production [29]. FBLN5 contributes to elastic fiber formation and, in a model of cutaneous fibrosis, has been implicated in a positive feedback loop linking matrix stiffness, inflammation, and further extracellular matrix secretion [30]. In contrast, SPARC contributes to type I procollagen processing and has been shown in vitro to inhibit the matrix metalloproteinase (MMP)-9-mediated degradation of type I and III collagens; it also directly binds fibrillar collagens and regulates their incorporation into the extracellular matrix [16,17,31]. SPARC can also exert opposing effects on fibrosis depending on its cellular source, as demonstrated in a mouse model of bleomycin-induced lung injury [15]. Thus, extrapolating from the higher plasma levels of OPN, OPN N-half, and FBLN5 to assume that SPARC would also increase in parallel with local fibrotic activity may have overlooked the tissue-specific behavior of SPARC.

### 3.3. The Hypothesis of Local Retention of SPARC

One possible explanation for the lower plasma SPARC concentrations in patients who developed SDHC is increased local binding and retention of SPARC within the collagen-rich meningeal extracellular matrix. SPARC recognizes specific sequences in fibrillar collagens and regulates type I procollagen’s processing and incorporation into the insoluble extracellular matrix [16,17]. The limited cleavage of SPARC by MMPs further increased its binding activity for types I, IV, and V collagen by approximately 7- to 20-fold [32]. In type I collagen-deficient Mov-13 mice, SPARC production and secretion were preserved, whereas its accumulation in tissues and insoluble extracellular matrix was markedly impaired [33]. These findings demonstrate that SPARC’s production and secretion can be dissociated from its retention within the extracellular matrix.

Applied to the present findings, a greater proportion of SPARC produced and secreted within intracranial tissues after SAH may have been retained within the collagen-rich meningeal extracellular matrix in patients who subsequently developed SDHC. Consequently, a smaller proportion may have entered the circulation and been detected by the assay, resulting in lower plasma concentrations. This hypothesis could account for both the involvement of SPARC in collagen formation and fibrotic remodeling and the lower plasma SPARC concentrations observed in the SDHC group.

However, this explanation remains purely speculative because neither CSF SPARC concentrations nor local SPARC retention were assessed in this study. Altered systemic SPARC production or clearance, platelet-related effects, treatment, and residual confounding are also possible explanations for the observed association. The present data do not distinguish these possibilities from local retention. The assay measured immunoreactive SPARC and did not separately quantify intact SPARC and specific proteolytic fragments. Equivalent detection of these molecular forms was not established. Experimental studies using SAH models are therefore needed to clarify the relationships among SPARC localization, molecular forms, extracellular matrix remodeling, and SDHC development.

### 3.4. Limitation

This study has several limitations. First, it was a retrospective analysis of prospectively collected data from a multicenter registry conducted in a single region of Japan. Unmeasured confounding cannot be excluded, and the observed associations do not establish causality. Patients with concomitant inflammatory diseases and bacterial infections were excluded from the main analysis to eliminate factors that could influence plasma SPARC concentrations. The exclusion of patients with unavailable SPARC measurements or SDHC follow-up data may also have introduced selection bias if data availability was related to disease severity or clinical course. The regional setting and these eligibility restrictions may affect the generalizability of the findings and limit their applicability to the broader population of patients with aneurysmal SAH. The direction and magnitude of any resulting selection bias remain uncertain. Infection severity and exact onset dates were unavailable, limiting assessment of infection-related effects and precluding adjustment for time-varying infection status. The small infection-site subgroups also limited the precision of those exploratory estimates. Inter-center variation in shunt placement decisions, which were made without a study-specific standardized protocol, may have influenced SDHC ascertainment, and cases developing after 90 days post-SAH may have been missed. Death may have limited the opportunity to observe SDHC, and its potential influence could not be fully assessed because exact death dates were unavailable. Second, SPARC was measured only in plasma, and paired CSF samples and meningeal tissue were unavailable. Therefore, the source of circulating SPARC and its relationships with intracranial collagen deposition, local tissue retention, and systemic processes could not be determined. Because of limited sample availability, the study-specific assay reproducibility and the potential effects of prolonged sample storage on measured SPARC concentrations could not be fully assessed. In addition, because no additional centrifugation was performed for platelet removal, residual platelets and ex vivo platelet activation during sample handling may have contributed to the measured plasma SPARC concentrations. Finally, the subgroup analyses and the post hoc multivariable logistic regression analysis using plasma SPARC concentrations at days 4–6 were exploratory. Given the nonsignificant SDHC-by-period interaction, these findings should be interpreted cautiously and regarded as hypothesis-generating. Although adding SPARC modestly increased the AUC and yielded a positive IDI estimate, the 95% CI for continuous NRI included zero. The clinical relevance of this modest improvement remains uncertain, and the incremental clinical value of plasma SPARC for SDHC prediction requires validation in independent cohorts.

## 4. Materials and Methods

### 4.1. Study Design and Patient Population

This study was a retrospective analysis using clinical data that had been prospectively collected in pSEED. The registry enrolled consecutive patients at nine tertiary referral centers in Mie Prefecture, Japan, between September 2013 and December 2016. Plasma samples in the registry were also collected at post-aneurysmal SAH days 1–3, 4–6, 7–9, and 10–12 after aneurysm obliteration. In the present study, previously measured plasma SPARC concentrations were used to examine their associations with clinical variables, particularly SDHC. Seventy-two of the 186 patients were also included in our previous study of plasma SPARC and DCI, which analyzed 117 patients [21].

The inclusion criteria were 20 years or older, pre-onset mRS 0–2, SAH confirmed by computed tomography (CT) or lumbar puncture, ruptured saccular intracranial aneurysms confirmed by CT angiography or digital subtraction angiography, and aneurysm obliteration by endovascular coiling or microsurgical clipping within 48 h after aneurysmal SAH onset. For the main analysis, the exclusion criteria were as follows: ruptured traumatic, dissecting, mycotic, and arteriovenous malformation-related aneurysms or SAH of unknown etiology; ruptured aneurysms treated by parent artery occlusion; chronic kidney disease present at admission; conditions that might affect plasma SPARC levels such as inflammatory diseases and malignancies present at admission, pre-admission use of immunosuppressants, and bacterial infections recorded during hospitalization; no available SPARC measurements; and missing SDHC status. Patients with known SDHC status and partially available serial SPARC measurements were retained.

After the diagnosis of aneurysmal SAH, treatment and management strategies were determined at the discretion of the attending neurosurgeons at each participating center, without study-specific restrictions. These strategies included the choice of treatment modality for aneurysm obliteration, the timing of aneurysm treatment, and the management of DCI, AVSP, DCIn, and other medical conditions.

This study adhered to the Strengthening the Reporting of Observational Studies in Epidemiology (STROBE) guidelines.

### 4.2. Clinical Variables

The baseline variables included age, sex, pre-onset mRS score, past medical history, comorbidity, smoking and alcohol use, family history of aneurysmal SAH, admission WFNS grade, modified Fisher grade [34], acute hydrocephalus, and ruptured aneurysm location. Treatment-related variables included aneurysm treatment modality, coiling/clipping-related complications, CSF drainage, and prophylactic anti-DCI medications. Clinical events included DCI, AVSP, DCIn, and SDHC. Functional outcome was assessed using the mRS at 90 days after aneurysmal SAH.

Acute hydrocephalus was defined as ventriculomegaly by CT scans on admission and was considered to cause impaired consciousness. All patients underwent CT or magnetic resonance (MR) imaging on the day after aneurysm obliteration. Coiling/clipping-related complications were defined as cerebral contusion and/or infarction identified on these images. CSF drainage included ventricular, cisternal, and spinal drainage performed to manage hydrocephalus, control intracranial pressure, or promote clearance of subarachnoid blood. DCI was defined as the delayed onset of focal neurological impairment (such as hemiparesis, aphasia, apraxia, hemianopia, or neglect) or a decrease of at least 2 points on the Glasgow Coma Scale (either the total score or its individual components [eye, motor on either side, or verbal]) lasting for at least 1 h, which was not evident immediately after aneurysm occlusion and was not determined to be attributed to other causes, including iatrogenic ones, by clinical assessment, CT scans, or MR imaging of the brain, as well as by the appropriate laboratory tests [35]. AVSP was defined as a reduction of more than 50% from the baseline vessel diameter in any of the following arteries: the intracranial internal carotid artery; the A1–A2, M1–M2, or P1–P2 segments of the anterior, middle, or posterior cerebral arteries, respectively; the vertebral artery; or the basilar artery, irrespective of the presence of clinical symptoms. DCIn was defined as a newly identified non-iatrogenic cerebral infarct on follow-up CT or MR imaging that had been absent on imaging obtained the day after aneurysm obliteration or another intervention. SDHC was defined as neurological deterioration attributable to progressive ventricular enlargement occurring after the first 14 days following SAH and requiring permanent CSF shunt placement. SDHC was recorded as an event until death or post-SAH 90 days. Decisions regarding CSF drainage weaning and permanent CSF shunt placement were made by the treating neurosurgeons at each center, without a study-specific standardized protocol. Permanent CSF shunt placement was confirmed by a review of the medical records. Neurological deterioration was assessed clinically without a study-specific quantitative threshold.

### 4.3. Blood Sample Collection, Processing, and Measurement

Blood samples were obtained from peripheral veins with minimal stasis in the early morning during post-onset days 1–3, 4–6, 7–9, and 10–12 after aneurysm obliteration. Immediately after collection, blood samples were placed in tubes containing a plasma separator and centrifuged at 3000 rpm for 5 min, and the separated plasma was stored at −78 °C until analysis [26]. The registry protocol for multiple biomarker measurements did not include an additional centrifugation step for platelet removal before freezing. Because the samples had already been frozen, this pre-freezing step could not be performed retrospectively. Experienced technicians blinded to the clinical data measured the plasma concentrations of immunoreactive SPARC in these samples using a commercially available enzyme-linked immunosorbent assay kit for Human SPARC (Code No. DSP00; R&D Systems, Minneapolis, MN, USA). The assay uses a quantitative sandwich format, with a manufacturer-reported minimum detectable dose of 0.044–0.269 ng/mL (mean, 0.099 ng/mL). All SPARC values used in this study were obtained from previous measurements; no new assays or re-assays were performed for the present study.

### 4.4. Statistical Analysis

Continuous variables are presented as medians with interquartile ranges. Categorical variables are presented as numbers and percentages, and ASDs were also calculated to describe between-group differences in baseline characteristics.

Plasma SPARC concentrations were analyzed using linear mixed-effects models. The inspection of residual histograms and Q–Q plots indicated that the residuals were not approximately normally distributed on the original scale; therefore, plasma SPARC concentrations were log-transformed before analysis. Sampling period was treated as a categorical variable with four levels: days 1–3, 4–6, 7–9, and 10–12. The unadjusted model included SDHC status, sampling period, and their interaction as fixed effects. The adjusted model additionally included age, admission WFNS grade V, modified Fisher grade 4, and acute hydrocephalus as fixed effects. Both models used all available SPARC measurements from the same cohort and included a patient-specific random intercept to account for within-patient correlations. Missing values were not imputed. Models were estimated using restricted maximum likelihood, and the SDHC-by-period interaction was assessed using a Kenward–Roger F test. Model-based estimated marginal means were back-transformed to provide geometric means. Between-group contrasts within each period are expressed as GMRs, calculated as SDHC divided by no SDHC, with pointwise 95% CIs and Holm-adjusted *p* values. The interaction *p* value is reported separately. As a sensitivity analysis, the unadjusted and adjusted mixed-effects models were refitted after excluding six patients with a 90-day mRS score of 6 and no recorded SDHC.

Exploratory subgroup analyses were performed according to age (<75 or ≥75 years), admission WFNS grades (I–IV or V), modified Fisher grades (1–3 or 4), acute hydrocephalus, treatment modality, and CSF drainage. A separate unadjusted log-scale mixed-effects model with a patient-specific random intercept was fitted within each subgroup.

Because the clearest between-group difference in the mixed-effects analysis was observed at days 4–6, a post hoc exploratory multivariable logistic regression analysis was performed to estimate the association between plasma SPARC concentrations at days 4–6 and the subsequent development of SDHC. The model was adjusted for age, admission WFNS grade V, modified Fisher grade 4, and acute hydrocephalus. aORs, 95% CIs, and *p* values are reported. The logistic regression analysis included patients with available SPARC measurements at days 4–6 and complete data on SDHC status and the included covariates. A sensitivity analysis was performed by additionally adjusting the logistic regression model for the use of any ventricular, cisternal, or spinal CSF drainage during hospitalization.

Clinical characteristics of the main analysis cohort and patients excluded due to bacterial infections were compared descriptively using ASDs. A separate sensitivity analysis refitted the adjusted mixed-effects and days 4–6 logistic regression models after including infection-excluded patients with available SPARC measurements and known SDHC status, retaining the four clinical covariates and adding bacterial infection recorded during hospitalization as a patient-level binary variable. Exploratory analyses among patients with infections used separate unadjusted log-scale mixed-effects models within CNS and non-CNS strata, each including SDHC status, sampling period, their interaction, and a patient-specific random intercept.

As an additional exploratory analysis in the main analysis cohort, the clinical logistic regression model containing age, admission WFNS grade V, modified Fisher grade 4, and acute hydrocephalus was compared with the same model additionally including the plasma SPARC concentration at days 4–6 as a continuous variable. Both models were fitted in the same 184 patients with complete data for the included variables. Discrimination was assessed using the AUC, and optimism-corrected AUCs were estimated using 1000 bootstrap resamples. Continuous (category-free) NRI and IDI were calculated using the model-development data without optimism correction, with 95% CIs based on a normal approximation. The 95% CIs for AUCs in the model-development data were calculated using the DeLong method. Approximate 95% CIs for optimism-corrected AUCs were obtained by transforming the confidence limits for Somers’ Dxy returned by the validate function in the rms package version 8.1-1 to the AUC scale.

All tests were two-sided, and *p* < 0.05 was considered statistically significant. *p* values for the four period-specific contrasts were adjusted using the Holm method separately within each model. The interaction *p* value was not included in these adjustment families. Analyses were performed using R software (version 4.6.1; R Foundation for Statistical Computing, Vienna, Austria).

### 4.5. Artificial Intelligence

ChatGPT (OpenAI, GPT-6 Pro) was used in part to assist with grammar, spelling, and sentence structure during manuscript preparation. All texts were reviewed and revised by the authors, who take full responsibility for the final content.

## 5. Conclusions

In this multicenter registry study, lower plasma SPARC concentrations, particularly at days 4–6 after aneurysmal SAH, were associated with the subsequent development of SDHC. Further clinical and experimental studies are needed to determine whether this association reflects altered SPARC localization or molecular processing during meningeal extracellular matrix remodeling.

## Figures and Tables

**Table 1:** Clinical variables according to shunt-dependent hydrocephalus status.

Variable	Overall (*n* = 186)	No SDHC (*n* = 132)	SDHC (*n* = 54)	ASD
Age (years), median (IQR)	65.5 (50.2–73.0)	60.0 (47.0–71.0)	72.0 (65.2–78.0)	0.802
Female	134 (72.0%)	89 (67.4%)	45 (83.3%)	0.376
Past medical history				
Cerebral infarction	10 (5.4%)	5 (3.8%)	5 (9.3%)	0.223
Comorbidity				
Hypertension	84 (45.2%)	56 (42.4%)	28 (51.9%)	0.190
Diabetes mellitus	11 (5.9%)	7 (5.3%)	4 (7.4%)	0.086
Dyslipidemia	19 (10.2%)	14 (10.6%)	5 (9.3%)	0.045
Smoking status				0.335
Current	43 (23.1%)	33 (25.0%)	10 (18.5%)	
Former	5 (2.7%)	5 (3.8%)	0 (0.0%)	
Never	138 (74.2%)	94 (71.2%)	44 (81.5%)	
Alcohol use	35 (18.8%)	26 (19.7%)	9 (16.7%)	0.079
Family history of SAH	20 (10.8%)	19 (14.4%)	1 (1.9%)	0.472
Pre-onset mRS score				0.375
0	172 (92.5%)	126 (95.5%)	46 (85.2%)	
1	11 (5.9%)	4 (3.0%)	7 (13.0%)	
2	3 (1.6%)	2 (1.5%)	1 (1.9%)	
Admission WFNS grade				0.719
I	57 (30.6%)	50 (37.9%)	7 (13.0%)	
II	52 (28.0%)	37 (28.0%)	15 (27.8%)	
III	10 (5.4%)	6 (4.5%)	4 (7.4%)	
IV	28 (15.1%)	20 (15.2%)	8 (14.8%)	
V	39 (21.0%)	19 (14.4%)	20 (37.0%)	
Modified Fisher grade				0.587
1	23 (12.4%)	21 (15.9%)	2 (3.7%)	
2	8 (4.3%)	7 (5.3%)	1 (1.9%)	
3	91 (48.9%)	67 (50.8%)	24 (44.4%)	
4	64 (34.4%)	37 (28.0%)	27 (50.0%)	
Acute hydrocephalus	63 (33.9%)	29 (22.0%)	34 (63.0%)	0.911
Ruptured aneurysm location				0.094
Anterior circulation	169 (90.9%)	121 (91.7%)	48 (88.9%)	
Posterior circulation	17 (9.1%)	11 (8.3%)	6 (11.1%)	
Treatment modality				0.104
Endovascular coiling	40 (21.5%)	30 (22.7%)	10 (18.5%)	
Microsurgical clipping	146 (78.5%)	102 (77.3%)	44 (81.5%)	
Coiling/clipping-related complication	48 (25.8%)	32 (24.2%)	16 (29.6%)	0.122
Cerebral contusion	7 (3.8%)	6 (4.5%)	1 (1.9%)	0.154
Cerebral infarction	41 (22.0%)	26 (19.7%)	15 (27.8%)	0.191
Cerebrospinal fluid drainage	65 (34.9%)	31 (23.5%)	34 (63.0%)	0.869
Ventricular	47 (25.3%)	16 (12.1%)	31 (57.4%)	1.081
Cisternal	23 (12.4%)	15 (11.4%)	8 (14.8%)	0.102
Spinal	20 (10.8%)	10 (7.6%)	10 (18.5%)	0.329
Prophylaxis for DCI				
Fasudil	181 (97.3%)	130 (98.5%)	51 (94.4%)	0.220
Cilostazol	150 (80.6%)	106 (80.3%)	44 (81.5%)	0.030
Eicosapentaenoic acid	93 (50.0%)	68 (51.5%)	25 (46.3%)	0.105
Statin	44 (23.7%)	34 (25.8%)	10 (18.5%)	0.175
DCI	30 (16.1%)	15 (11.4%)	15 (27.8%)	0.423
Angiographic vasospasm	61 (32.8%)	35 (26.5%)	26 (48.1%)	0.459
Delayed cerebral infarction	44 (23.7%)	26 (19.7%)	18 (33.3%)	0.313
mRS score at 90 days				1.543
0	66 (35.5%)	64 (48.5%)	2 (3.7%)	
1	25 (13.4%)	21 (15.9%)	4 (7.4%)	
2	25 (13.4%)	17 (12.9%)	8 (14.8%)	
3	17 (9.1%)	9 (6.8%)	8 (14.8%)	
4	15 (8.1%)	5 (3.8%)	10 (18.5%)	
5	28 (15.1%)	9 (6.8%)	19 (35.2%)	
6	8 (4.3%)	6 (4.5%)	2 (3.7%)	
Missing	2 (1.1%)	1 (0.8%)	1 (1.9%)	
mRS score 0–2 at 90 days, n/N (%)	116/184 (63.0%)	102/131 (77.9%)	14/53 (26.4%)	1.201

Data are numbers of patients (% of total patients per group) unless otherwise specified. For entries presented as n/N (%), N denotes the number of patients with available data in each group. Missing data are reported separately. ASDs are calculated using non-missing data for each variable and are reported as absolute values; larger values indicate greater between-group differences. Ventricular, cisternal, and spinal drainage categories are not mutually exclusive. ASD, absolute standardized difference; DCI, delayed cerebral ischemia; IQR, interquartile range; mRS, modified Rankin Scale; SAH, subarachnoid hemorrhage; SDHC, shunt-dependent hydrocephalus; WFNS, World Federation of Neurological Surgeons.

**Table 2:** Post hoc multivariable logistic regression analysis of the association between plasma SPARC concentrations at days 4–6 and shunt-dependent hydrocephalus.

Variable	aOR (95% CI)	*p* Value
Acute hydrocephalus	3.74 (1.71 to 8.17)	<0.001
Admission WFNS grade V	2.84 (1.14 to 7.11)	0.026
Age, per 10-year increase	1.47 (1.11 to 1.95)	0.008
Modified Fisher grade 4	1.18 (0.52 to 2.68)	0.691
Plasma SPARC concentration at days 4–6, per 10 ng/mL increase	0.91 (0.84 to 0.98)	0.011

aORs for age and plasma SPARC concentrations are expressed per 10-year and 10 ng/mL increase, respectively. Reference categories are no acute hydrocephalus, WFNS grades I–IV, and modified Fisher grades 1–3. aOR, adjusted odds ratio; CI, confidence interval; SPARC, secreted protein acidic and rich in cysteine; WFNS, World Federation of Neurological Surgeons.

## Data Availability

The data presented in this study are available on request from the corresponding author due to ethical restrictions.

## Supplementary Materials

The following supporting information can be downloaded at: https://www.mdpi.com/article/10.3390/ijms27219929/s1.

## Abbreviations

The following abbreviations are used in this manuscript:

aOR	Adjusted odds ratio
ASD	Absolute standardized difference
AUC	Area under the receiver operating characteristic curve
AVSP	Angiographic vasospasm
CI	Confidence interval
CNS	Central nervous system
CSF	Cerebrospinal fluid
CT	Computed tomography
DCI	Delayed cerebral ischemia
DCIn	Delayed cerebral infarction
FBLN5	Fibulin-5
GMR	Geometric mean ratio
IDI	Integrated discrimination improvement
MMP	Matrix metalloproteinase
MR	Magnetic resonance
mRS	Modified Rankin Scale
NRI	Net reclassification improvement
OPN	Osteopontin
pSEED	Prospective Registry for Searching Mediators of Neurovascular Events After Aneurysmal SAH
ROC	Receiver operating characteristic
SAH	Subarachnoid hemorrhage
SDHC	Shunt-dependent hydrocephalus
SPARC	Secreted protein acidic and rich in cysteine
STROBE	Strengthening the Reporting of Observational Studies in Epidemiology
TGF-β	Transforming growth factor beta
WFNS	World Federation of Neurological Surgeons

## Appendix A

pSEED (Prospective Registry for Searching Mediators of Neurovascular Events After Aneurysmal Subarachnoid Hemorrhage) group: members along with their affiliations.

Department of Neurosurgery, Kuwana City Medical Center, Kuwana (Yasuyuki Umeda, Yoichi Miura, and Kazuaki Aoki); Mie Prefectural General Medical Center, Yokkaichi (Yusuke Kamei, and Takanori Sano); Suzuka Kaisei Hospital, Suzuka (Tomohiro Araki, Masato Shiba, and Naoki Ichikawa); Suzuka Chuo General Hospital, Suzuka (Shigetoshi Shimizu, Takuro Tsuchiya, and Reona Asada); Mie University Graduate School of Medicine, Tsu (Hidenori Suzuki, Naoki Toma, Ryuta Yasuda, Katsuhiro Tanaka, Fumihiro Kawakita, Hirofumi Nishikawa, Hideki Kanamaru, Yotaro Kitano, Yume Suzuki, Hideki Nakajima, and Koichi Hakozaki); Mie Chuo Medical Center, Tsu (Fujimaro Ishida, and Keiji Fukazawa); Matsusaka Chuo General Hospital, Matsusaka (Kazuhiko Tsuda, and Yu Sato); Saiseikai Matsusaka General Hospital, Matsusaka (Seiji Hatazaki, Kazuhide Hamada, and Mai Nampei); and Ise Red Cross Hospital, Ise (Fumitaka Miya, Hiroshi Tanemura, and Masashi Fujimoto).
