# Supplementary material for: Plasma SPARC Concentrations in Patients with Subsequent Shunt-Dependent Hydrocephalus After Aneurysmal Subarachnoid Hemorrhage
Source: Int J Mol Sci. 2026 Nov 6;27(21):9929. doi: 10.3390/ijms27219929 (PMC13763721; doi:10.3390/ijms27219929)
Supplement: Supplementary file 1 [file ijms-27-09929-s001.zip › ijms-4575123-supplementary.pdf]

Plasma SPARC Concentrations in Patients with Subsequent Shunt-Dependent Hydrocephalus After Aneurysmal Subarachnoid Hemorrhage

International Journal of Molecular Sciences

Koichi Hakozaki, Hideki Nakajima, Yotaro Kitano, Hirofumi Nishikawa, Fumihiro Kawakita, Ryuta Yasuda, Hidenori Suzuki, and pSEED group

Address Correspondence to: Hidenori Suzuki, MD, PhD

Department of Neurosurgery, Mie University Graduate School of Medicine,

2-174 Edobashi, Tsu, Mie 514-8507, Japan

E-mail: suzuki02@med.mie-u.ac.jp

Supplementary Figures S1−S4 and Tables S1−S10

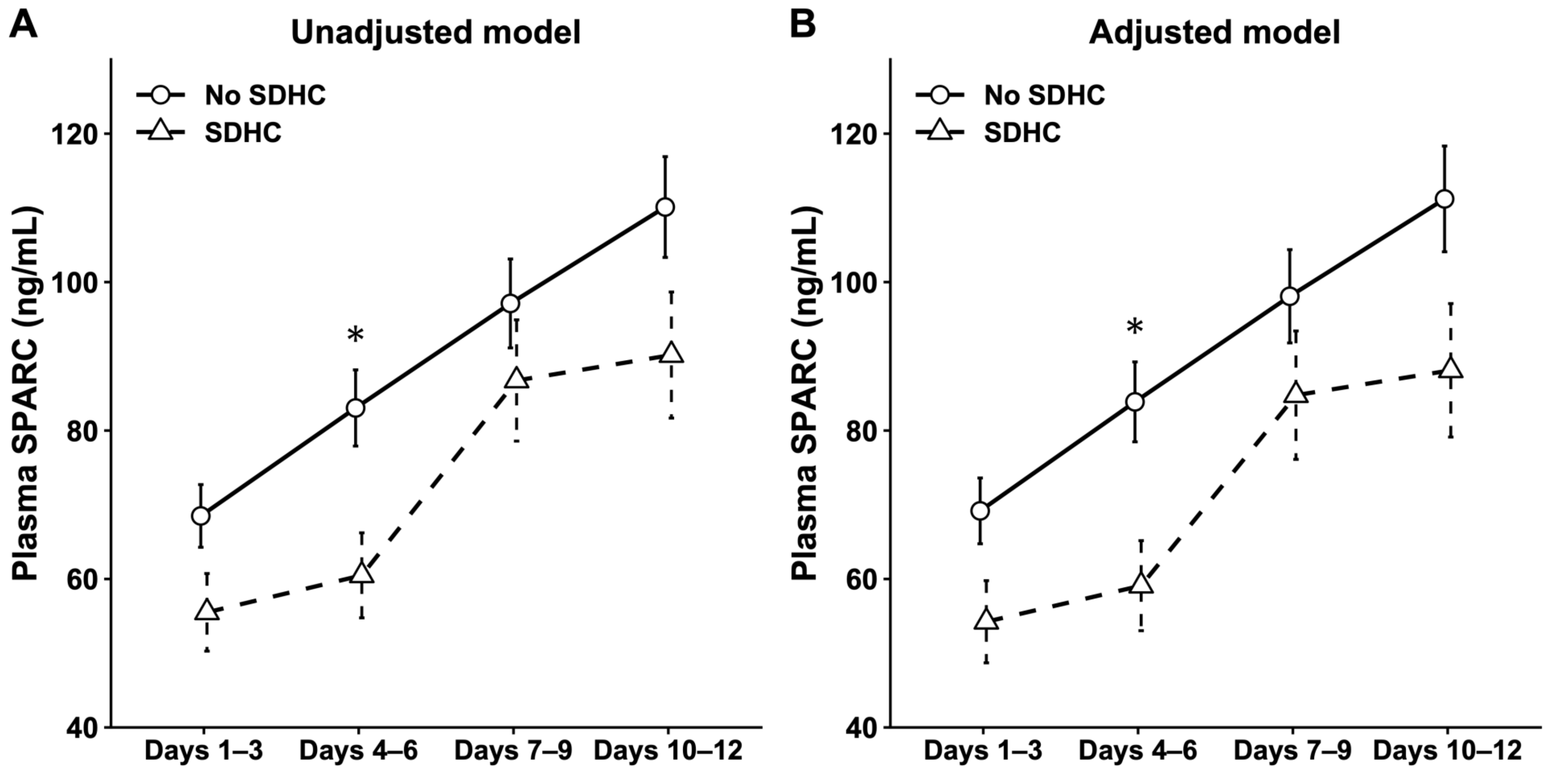

Supplementary Figure S1. Sensitivity analysis of temporal changes in plasma secreted protein acidic and rich in cysteine (SPARC) concentrations according to subsequent development of shunt-dependent hydrocephalus (SDHC) after aneurysmal subarachnoid hemorrhage. This analysis excludes six patients with a modified Rankin Scale (mRS) score of 6 at 90 days and no recorded SDHC. Model-estimated plasma SPARC concentrations are shown for patients who did and did not subsequently develop SDHC. Numbers of patients with available SPARC measurements at each sampling period are provided in Supplementary Table S3. (**A**) Unadjusted model. (**B**) Model adjusted for age, admission World Federation of Neurological Surgeons grade V, modified Fisher grade 4, and acute hydrocephalus. Both models include SDHC status, sampling period, and their interaction as fixed effects and a patient-specific random intercept. Plasma SPARC concentrations were log-transformed before analysis. Back-transformed estimated marginal means are presented as geometric means, and error bars indicate standard errors. *Holm-adjusted $P < 0.05$ for the between-group contrast within the corresponding sampling period.

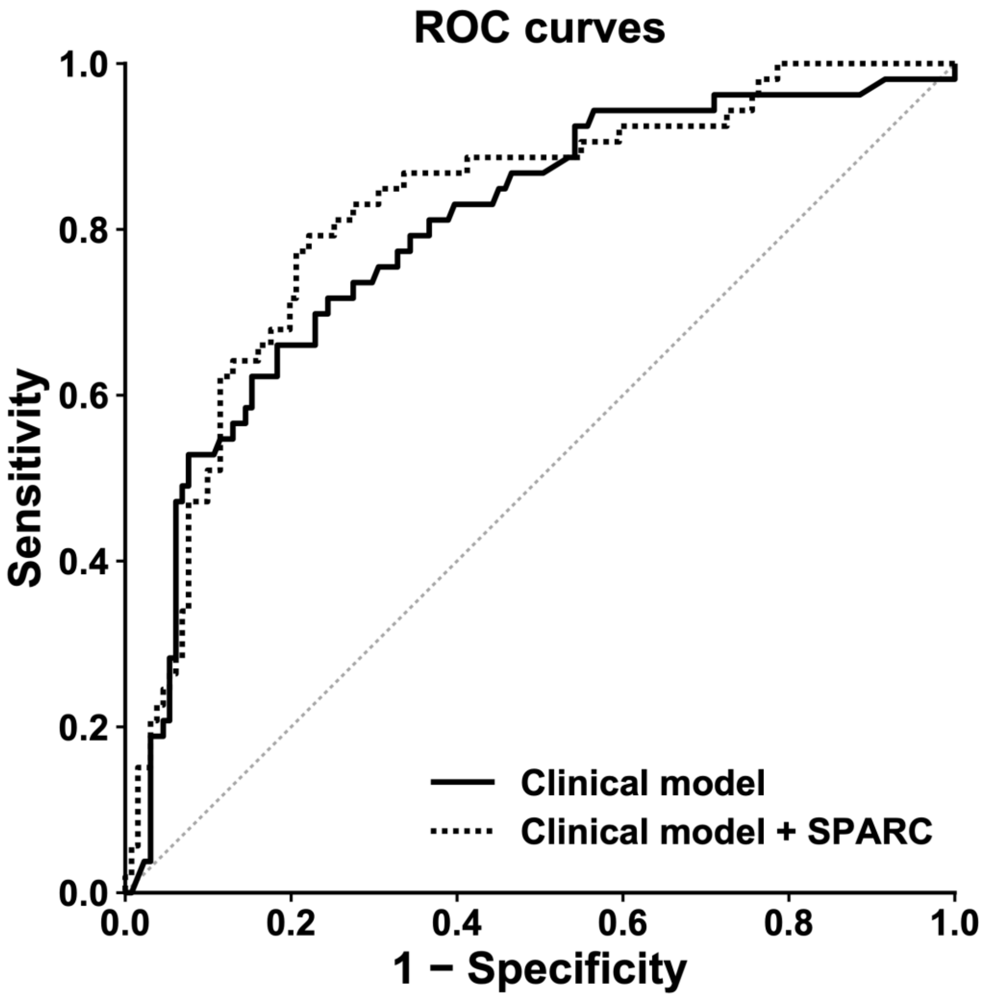

Supplementary Figure S2. Receiver operating characteristic (ROC) curves for shunt-dependent hydrocephalus using clinical models with and without plasma secreted protein acidic and rich in cysteine (SPARC). The solid line represents the clinical model including age, admission World Federation of Neurological Surgeons grade V, modified Fisher grade 4, and acute hydrocephalus. The dotted line represents the same model with the addition of plasma SPARC concentration at days 4–6. Both models are fitted and evaluated in the same 184 patients; the curves therefore represent performance in the model-development data. The gray diagonal line indicates chance-level discrimination. Areas under the ROC curve (AUCs) and bootstrap optimism-corrected AUCs, with their 95% confidence intervals, are provided in Supplementary Table S6.

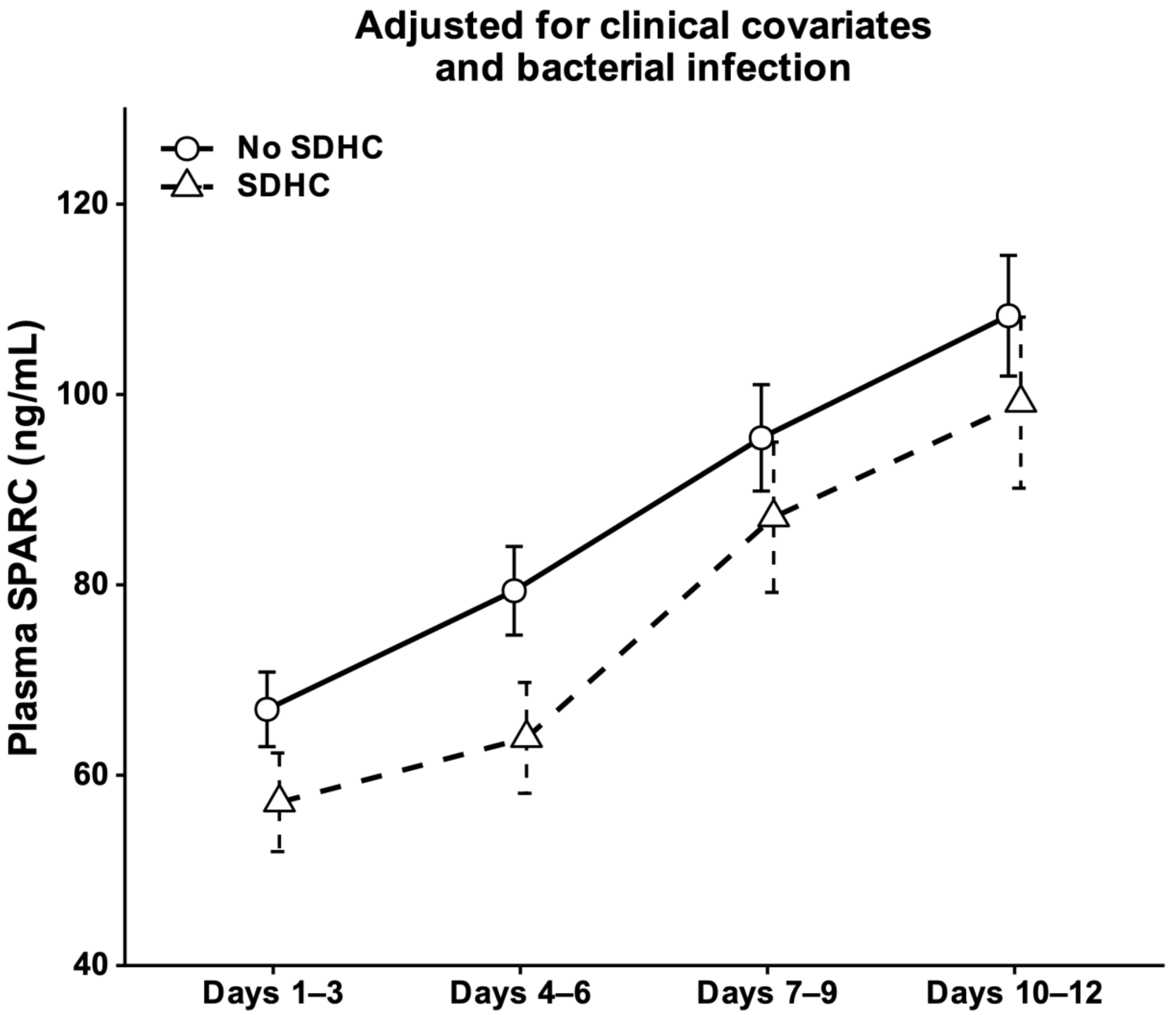

Supplementary Figure S3. Sensitivity analysis of temporal changes in plasma secreted protein acidic and rich in cysteine (SPARC) concentrations according to subsequent development of shunt-dependent hydrocephalus (SDHC) after aneurysmal subarachnoid hemorrhage. This analysis includes 29 patients previously excluded due to bacterial infections, giving a total of 215 patients. Model-estimated plasma SPARC concentrations are shown for patients who did and did not subsequently develop SDHC. Numbers of patients with available SPARC measurements at each sampling period are provided in Supplementary Table S8. The model includes SDHC status, sampling period, and their interaction as fixed effects and a patient-specific random intercept, with adjustment for age, admission World Federation of Neurological Surgeons grade V, modified Fisher grade 4, acute hydrocephalus, and bacterial infection. Plasma SPARC concentrations were log-transformed before analysis. Back-transformed estimated marginal means are presented as geometric means, and error bars indicate standard errors.

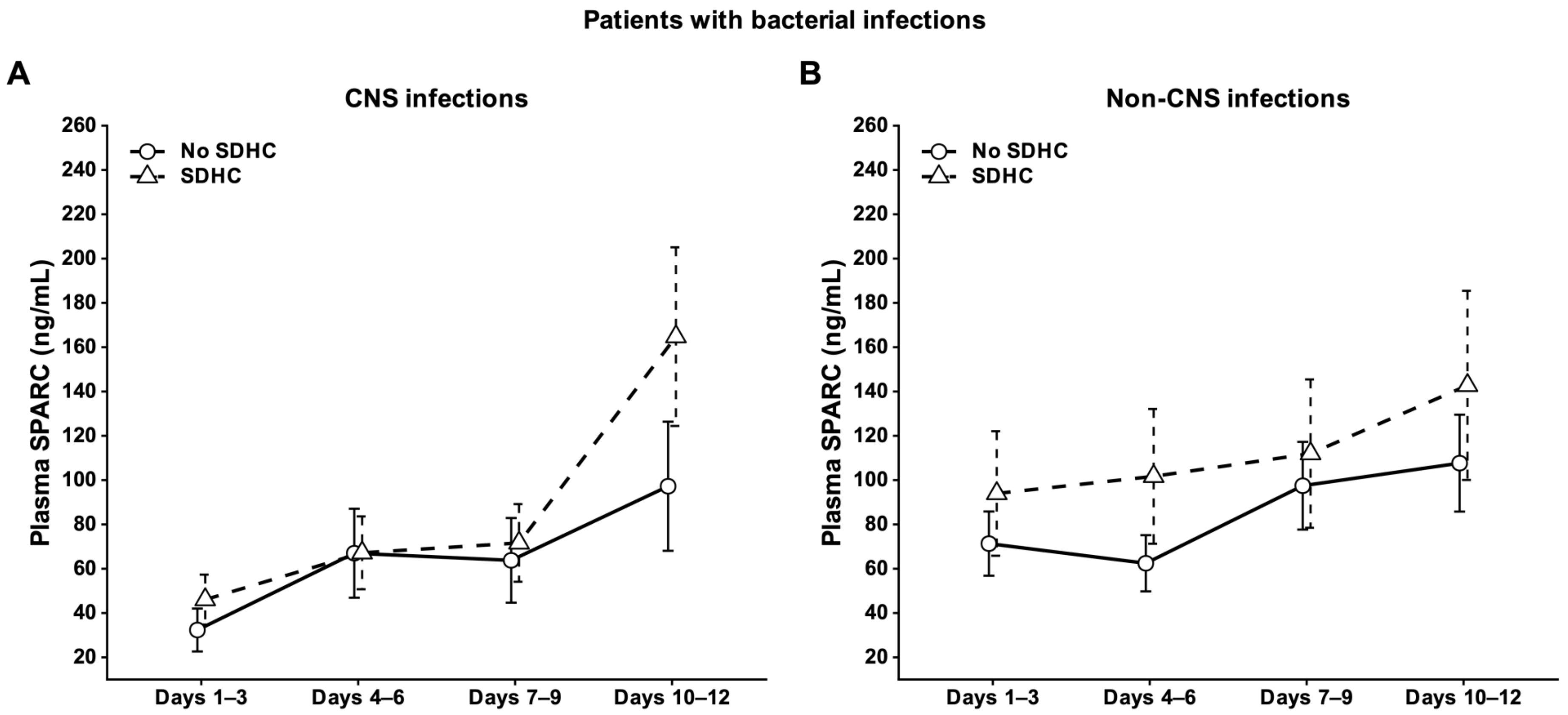

Supplementary Figure S4. Exploratory analyses of temporal changes in plasma secreted protein acidic and rich in cysteine (SPARC) concentrations according to shunt-dependent hydrocephalus (SDHC) status in patients with bacterial infections, stratified by infection sites. (**A**) Central nervous system (CNS) infections. (**B**) Non-CNS infections. Each panel is based on a separate unadjusted linear mixed-effects model that includes SDHC status, sampling period, and their interaction as fixed effects and a patient-specific random intercept. Plasma SPARC concentrations were log-transformed before analysis. Back-transformed estimated marginal means are presented as geometric means, and error bars indicate standard errors. Numbers of patients with available measurements and period-specific geometric mean ratios are provided in Supplementary Table S9. Holm adjustment is applied across the four period-specific contrasts separately within each infection-site stratum. Infection site refers to infection recorded during hospitalization, not infection status at each sampling time.

Supplementary Table S1. Model-estimated plasma secreted protein acidic and rich in cysteine concentrations according to shunt-dependent hydrocephalus (SDHC) status.

Model	Sampling period	No SDHC/SDHC, n	No SDHC, GM (95% CI)	SDHC, GM (95% CI)
Unadjusted	Days 1–3	132/54	67.9 (60.4 to 76.4)	55.5 (46.1 to 66.8)
	Days 4–6	131/53	81.6 (72.5 to 91.9)	60.5 (50.2 to 72.8)
	Days 7–9	132/54	96.3 (85.6 to 108.4)	86.7 (72.1 to 104.3)
	Days 10–12	132/54	108.6 (96.5 to 122.2)	90.2 (75.0 to 108.5)
Adjusted	Days 1–3	132/54	68.3 (60.4 to 77.2)	54.8 (44.9 to 66.8)
	Days 4–6	131/53	82.1 (72.6 to 92.8)	59.7 (48.9 to 72.8)
	Days 7–9	132/54	96.9 (85.7 to 109.4)	85.6 (70.2 to 104.3)
	Days 10–12	132/54	109.2 (96.6 to 123.4)	89.0 (73.0 to 108.5)

Values are back-transformed estimated marginal means from the log-scale mixed-effects models and are presented as geometric means (GMs) with pointwise 95% confidence intervals (CIs). n denotes the number of patients with available measurements at each sampling period, presented as No SDHC/SDHC. The adjusted model includes age, admission World Federation of Neurological Surgeons grade V, modified Fisher grade 4, and acute hydrocephalus. Values are expressed in ng/mL.

Supplementary Table S2. Period-specific geometric mean ratios (GMRs) of plasma secreted protein acidic and rich in cysteine (SPARC) concentrations according to shunt-dependent hydrocephalus (SDHC) status.

Sampling period	No SDHC/SDHC, n	Unadjusted GMR (95% CI)	Holm-adjusted P value	Adjusted GMR (95% CI)	Holm-adjusted P value
Days 1–3	132/54	0.82 (0.66 to 1.02)	0.213	0.80 (0.63 to 1.02)	0.218
Days 4–6	131/53	0.74 (0.59 to 0.92)	0.031	0.73 (0.57 to 0.93)	0.040
Days 7–9	132/54	0.90 (0.72 to 1.12)	0.348	0.88 (0.69 to 1.12)	0.314
Days 10–12	132/54	0.83 (0.67 to 1.03)	0.213	0.81 (0.64 to 1.04)	0.218

Plasma SPARC concentrations were log-transformed before mixed-effects modeling. n denotes the number of patients with available measurements at each sampling period, presented as No SDHC/SDHC. The adjusted model includes age, admission World Federation of Neurological Surgeons grade V, modified Fisher grade 4, and acute hydrocephalus. GMRs are calculated as SDHC/No SDHC; values below 1 indicate lower model-estimated plasma SPARC concentrations in the SDHC group. The 95% confidence intervals (CIs) are pointwise and are not adjusted for multiplicity. P values are adjusted using the Holm method across the four period-specific contrasts separately within each model.

Supplementary Table S3. Sensitivity analysis of period-specific geometric mean ratios (GMRs) of plasma secreted protein acidic and rich in cysteine (SPARC) concentrations according to shunt-dependent hydrocephalus (SDHC) status.

Sampling period	No SDHC/SDHC, n	Unadjusted GMR (95% CI)	Holm-adjusted P value	Adjusted GMR (95% CI)	Holm-adjusted P value
Days 1–3	126/54	0.81 (0.65 to 1.01)	0.188	0.78 (0.61 to 1.00)	0.161
Days 4–6	125/53	0.73 (0.58 to 0.91)	0.021	0.70 (0.55 to 0.90)	0.023
Days 7–9	126/54	0.89 (0.72 to 1.11)	0.316	0.86 (0.67 to 1.11)	0.246
Days 10–12	126/54	0.82 (0.66 to 1.02)	0.188	0.79 (0.62 to 1.01)	0.161

This analysis excludes six patients with a modified Rankin Scale score of 6 at 90 days and no recorded SDHC. Plasma SPARC concentrations were log-transformed before mixed-effects modeling. n denotes the number of patients with available measurements at each sampling period, presented as No SDHC/SDHC. The adjusted model includes age, admission World Federation of Neurological Surgeons grade V, modified Fisher grade 4, and acute hydrocephalus. GMRs are calculated as SDHC/No SDHC; values below 1 indicate lower model-estimated plasma SPARC concentrations in the SDHC group. The 95% confidence intervals (CIs) are pointwise and are not adjusted for multiplicity. P values are adjusted using the Holm method across the four period-specific contrasts separately within each model.

Supplementary Table S4. Period-specific geometric mean ratios (GMRs) of plasma secreted protein acidic and rich in cysteine (SPARC) concentrations according to shunt-dependent hydrocephalus (SDHC) status in exploratory subgroup analyses.

Subgroup	No SDHC/SDHC, n	Sampling period	Unadjusted GMR (95% CI)	Holm-adjusted P value	Interaction P value
Age <75 years					0.620
	109/33	Days 1–3	0.78 (0.59 to 1.03)	0.224	
	108/32	Days 4–6	0.75 (0.57 to 0.99)	0.172	
	109/33	Days 7–9	0.89 (0.67 to 1.17)	0.390	
	109/33	Days 10–12	0.83 (0.63 to 1.09)	0.347	
Age ≥75 years					0.275
	23/21	Days 1–3	0.96 (0.66 to 1.40)	1.000	
	23/21	Days 4–6	0.75 (0.52 to 1.10)	0.552	
	23/21	Days 7–9	1.03 (0.71 to 1.50)	1.000	
	23/21	Days 10–12	0.95 (0.65 to 1.39)	1.000	
Admission WFNS grades I–IV					0.162
	113/34	Days 1–3	0.85 (0.65 to 1.10)	0.667	
	112/33	Days 4–6	0.78 (0.60 to 1.02)	0.273	
	113/34	Days 7–9	1.04 (0.80 to 1.35)	0.767	
	113/34	Days 10–12	0.88 (0.68 to 1.15)	0.705	
Admission WFNS grade V					0.517
	19/20	Days 1–3	0.72 (0.46 to 1.13)	0.147	
	19/20	Days 4–6	0.54 (0.34 to 0.85)	0.034	
	19/20	Days 7–9	0.58 (0.37 to 0.92)	0.063	
	19/20	Days 10–12	0.62 (0.39 to 0.98)	0.080	
Modified Fisher grades 1–3					0.675
	95/27	Days 1–3	0.85 (0.63 to 1.16)	1.000	
	94/26	Days 4–6	0.84 (0.61 to 1.14)	1.000	
	95/27	Days 7–9	0.97 (0.71 to 1.32)	1.000	
	95/27	Days 10–12	0.84 (0.62 to 1.15)	1.000	
Modified Fisher grade 4					0.411
	37/27	Days 1–3	0.81 (0.59 to 1.12)	0.401	
	37/27	Days 4–6	0.63 (0.46 to 0.87)	0.023	
	37/27	Days 7–9	0.78 (0.57 to 1.08)	0.401	
	37/27	Days 10–12	0.80 (0.58 to 1.11)	0.401	
No acute hydrocephalus					0.614
	103/20	Days 1–3	0.74 (0.52 to 1.04)	0.336	
	102/19	Days 4–6	0.74 (0.52 to 1.06)	0.336	

	103/20	Days 7–9	0.86 (0.61 to 1.21)	0.767	
	103/20	Days 10–12	0.87 (0.62 to 1.24)	0.767	
Acute hydrocephalus					0.407
	29/34	Days 1–3	0.85 (0.62 to 1.16)	0.610	
	29/34	Days 4–6	0.78 (0.57 to 1.07)	0.374	
	29/34	Days 7–9	0.93 (0.68 to 1.28)	0.655	
	29/34	Days 10–12	0.73 (0.53 to 1.01)	0.222	
Coiling					0.897
	30/10	Days 1–3	0.76 (0.46 to 1.23)	0.439	
	30/10	Days 4–6	0.61 (0.37 to 1.00)	0.194	
	30/10	Days 7–9	0.72 (0.44 to 1.18)	0.439	
	30/10	Days 10–12	0.70 (0.43 to 1.14)	0.439	
Clipping					0.329
	102/44	Days 1–3	0.82 (0.64 to 1.05)	0.344	
	101/43	Days 4–6	0.77 (0.60 to 0.98)	0.129	
	102/44	Days 7–9	0.93 (0.73 to 1.19)	0.578	
	102/44	Days 10–12	0.86 (0.67 to 1.09)	0.431	
No cerebrospinal fluid drainage					0.637
	101/20	Days 1–3	0.80 (0.57 to 1.12)	0.570	
	100/19	Days 4–6	0.76 (0.54 to 1.07)	0.452	
	101/20	Days 7–9	0.89 (0.64 to 1.24)	0.971	
	101/20	Days 10–12	0.91 (0.65 to 1.28)	0.971	
Cerebrospinal fluid drainage					0.141
	31/34	Days 1–3	0.87 (0.62 to 1.22)	0.815	
	31/34	Days 4–6	0.73 (0.52 to 1.02)	0.188	
	31/34	Days 7–9	0.89 (0.64 to 1.25)	0.815	
	31/34	Days 10–12	0.66 (0.47 to 0.92)	0.059	

Plasma SPARC concentrations were log-transformed before mixed-effects modeling. Each subgroup is analyzed using a separate unadjusted mixed-effects model. n indicates the number of patients with available SPARC measurements in each group (No SDHC/SDHC) at each sampling period within each subgroup. GMRs are calculated as SDHC/No SDHC; values below 1 indicate lower model-estimated plasma SPARC concentrations in the SDHC group. The 95% confidence intervals (CIs) are pointwise and are not adjusted for multiplicity. P values are adjusted using the Holm method across the four period-specific contrasts within each subgroup. Interaction P values refer to the SDHC-by-period interaction within each subgroup and are not included in the Holm adjustment. WFNS, World Federation of Neurological Surgeons.

Supplementary Table S5. Post hoc multivariable logistic regression analysis of the association between plasma secreted protein acidic and rich in cysteine (SPARC) concentrations at days 4–6 and shunt-dependent hydrocephalus: sensitivity analysis with additional adjustment for cerebrospinal fluid drainage.

Variable	aOR (95% CI)	P value
Cerebrospinal fluid drainage	4.87 (2.01 to 11.80)	<0.001
Admission WFNS grade V	2.65 (1.00 to 6.98)	0.049
Acute hydrocephalus	1.87 (0.76 to 4.61)	0.174
Age, per 10-year increase	1.64 (1.21 to 2.23)	0.002
Modified Fisher grade 4	1.20 (0.51 to 2.85)	0.678
Plasma SPARC concentration at days 4–6, per 10 ng/mL increase	0.90 (0.84 to 0.98)	0.012

Cerebrospinal fluid drainage denotes the use of any ventricular, cisternal, or spinal drainage. Adjusted odds ratios (aORs) for age and plasma SPARC concentrations are expressed per 10-year and 10 ng/mL increase, respectively. Reference categories are no cerebrospinal fluid drainage, World Federation of Neurological Surgeons (WFNS) grades I–IV, no acute hydrocephalus, and modified Fisher grades 1–3. CI, confidence interval.

Supplementary Table S6. Exploratory comparison of clinical models with and without plasma secreted protein acidic and rich in cysteine (SPARC) for shunt-dependent hydrocephalus.

	**Clinical model**	**Clinical model + SPARC**
AUC (95% CI)	0.796 (0.723 to 0.869)	0.821 (0.754 to 0.889)
Bootstrap optimism-corrected AUC (95% CI)	0.781 (0.713 to 0.856)	0.803 (0.739 to 0.873)
NRI (95% CI)	—	0.267 (-0.040 to 0.574)
IDI (95% CI)	—	0.037 (0.010 to 0.063)

Both logistic regression models are fitted in the same 184 patients with complete data for the included variables. The clinical model includes age, admission World Federation of Neurological Surgeons grade V, modified Fisher grade 4, and acute hydrocephalus. The clinical model + SPARC additionally include plasma SPARC concentration at days 4–6 as a continuous variable. Areas under the receiver operating characteristic curve (AUCs) in the first row, continuous (category-free) net reclassification improvement (NRI), and integrated discrimination improvement (IDI) are calculated using the model-development data. NRI and IDI compare the clinical model + SPARC with the clinical model as the reference. Optimism-corrected AUCs are estimated using 1,000 bootstrap resamples. Estimates are presented with 95% confidence intervals (CIs); those for optimism-corrected AUCs are approximate.

Supplementary Table S7. Clinical characteristics of patients selected for the main analysis cohort and those excluded due to bacterial infections.

Variable	Main analysis cohort (n = 186)	Excluded due to bacterial infection (n = 29)	ASD
Age (years), median (IQR)	65.5 (50.2–73.0)	69.0 (61.0–76.0)	0.450
Female	134 (72.0%)	19 (65.5%)	0.141
Past medical history			
Cerebral infarction	10 (5.4%)	0 (0.0%)	0.337
Comorbidity			
Hypertension	84 (45.2%)	15 (51.7%)	0.132
Diabetes mellitus	11 (5.9%)	3 (10.3%)	0.163
Dyslipidemia	19 (10.2%)	5 (17.2%)	0.205
Smoking status			0.070
Current	43 (23.1%)	6 (20.7%)	
Former	5 (2.7%)	1 (3.4%)	
Never	138 (74.2%)	22 (75.9%)	
Alcohol use	35 (18.8%)	6 (20.7%)	0.047
Family history of SAH	20 (10.8%)	0 (0.0%)	0.491
Pre-onset mRS score			0.126
0	172 (92.5%)	26 (89.7%)	
1	11 (5.9%)	2 (6.9%)	
2	3 (1.6%)	1 (3.4%)	
Admission WFNS grade			0.340
I	57 (30.6%)	8 (27.6%)	
II	52 (28.0%)	6 (20.7%)	
III	10 (5.4%)	2 (6.9%)	
IV	28 (15.1%)	3 (10.3%)	
V	39 (21.0%)	10 (34.5%)	
Modified Fisher grade			0.579
1	23 (12.4%)	0 (0.0%)	
2	8 (4.3%)	2 (6.9%)	
3	91 (48.9%)	13 (44.8%)	
4	64 (34.4%)	14 (48.3%)	
Acute hydrocephalus	63 (33.9%)	12 (41.4%)	0.155
Ruptured aneurysm location			0.236
Anterior circulation	169 (90.9%)	28 (96.6%)	
Posterior circulation	17 (9.1%)	1 (3.4%)	
Treatment modality			0.020
Endovascular coiling	40 (21.5%)	6 (20.7%)	
Microsurgical clipping	146 (78.5%)	23 (79.3%)	
Coiling/clipping-related complication	48 (25.8%)	7 (24.1%)	0.039
Cerebral contusion	7 (3.8%)	1 (3.4%)	0.017
Cerebral infarction	41 (22.0%)	6 (20.7%)	0.033
Cerebrospinal fluid drainage	65 (34.9%)	13 (44.8%)	0.203

Ventricular	47 (25.3%)	11 (37.9%)	0.275
Cisternal	23 (12.4%)	4 (13.8%)	0.042
Spinal	20 (10.8%)	3 (10.3%)	0.013
Prophylaxis for DCI			
Fasudil	181 (97.3%)	27 (93.1%)	0.198
Cilostazol	150 (80.6%)	27 (93.1%)	0.375
Eicosapentaenoic acid	93 (50.0%)	17 (58.6%)	0.174
Statin	44 (23.7%)	8 (27.6%)	0.090
DCI	30 (16.1%)	7 (24.1%)	0.201
Angiographic vasospasm	61 (32.8%)	6 (20.7%)	0.276
Delayed cerebral infarction	44 (23.7%)	8 (27.6%)	0.090
SDHC	54 (29.0%)	12 (41.4%)	0.261
mRS score at 90 days			0.632
0	66 (35.5%)	6 (20.7%)	
1	25 (13.4%)	2 (6.9%)	
2	25 (13.4%)	5 (17.2%)	
3	17 (9.1%)	1 (3.4%)	
4	15 (8.1%)	7 (24.1%)	
5	28 (15.1%)	5 (17.2%)	
6	8 (4.3%)	2 (6.9%)	
Missing	2 (1.1%)	1 (3.4%)	
mRS score 0–2 at 90 days, n/N (%)	116/184 (63.0%)	13/28 (46.4%)	0.339

Data are numbers of patients (% of all patients per group) unless otherwise specified. For entries presented as n/N (%), N denotes the number of patients with available data in each group. Missing data are reported separately. Absolute standardized differences (ASDs) are calculated using non-missing data and are reported as absolute values; larger values indicate greater between-group differences. Ventricular, cisternal, and spinal drainage categories are not mutually exclusive. Twenty-nine of the 30 patients primarily excluded due to bacterial infections had available secreted protein acidic and rich in cysteine measurements and known shunt-dependent hydrocephalus (SDHC) status; one patient with pneumonia lacked both. Among all 30 excluded patients, central nervous system (CNS) infections comprised meningitis (n = 9) and brain abscess (n = 1), and non-CNS infections comprised pneumonia (n = 18), puncture-site infection (n = 1), and thigh abscess (n = 1). The analyzed infection cohort includes 10 CNS and 19 non-CNS cases. Infections were recorded during hospitalization. DCI, delayed cerebral ischemia; IQR, interquartile range; mRS, modified Rankin Scale; SAH, subarachnoid hemorrhage; WFNS, World Federation of Neurological Surgeons.

Supplementary Table S8. Sensitivity analysis of period-specific geometric mean ratios (GMRs) of plasma secreted protein acidic and rich in cysteine (SPARC) concentrations according to shunt-dependent hydrocephalus (SDHC) status, including patients previously excluded due to bacterial infections, with additional adjustment for bacterial infection.

Sampling period	No SDHC/SDHC, n	Adjusted GMR (95% CI)	Holm-adjusted P value
Days 1–3	149/66	0.85 (0.69 to 1.06)	0.472
Days 4–6	148/65	0.81 (0.65 to 1.00)	0.214
Days 7–9	149/66	0.91 (0.73 to 1.14)	0.824
Days 10–12	149/66	0.92 (0.74 to 1.14)	0.824

The model includes 215 patients (including 29 patients with bacterial infections) and 858 measurements. Plasma SPARC concentrations were log-transformed before mixed-effects modeling. n denotes the number of patients with available measurements at each sampling period, presented as No SDHC/SDHC. The adjusted model includes age, admission World Federation of Neurological Surgeons grade V, modified Fisher grade 4, acute hydrocephalus, and bacterial infection. GMRs are calculated as SDHC/No SDHC; values below 1 indicate lower model-estimated plasma SPARC concentrations in the SDHC group. The 95% confidence intervals (CIs) are pointwise and are not adjusted for multiplicity. P values are adjusted using the Holm method across the four period-specific contrasts. The SDHC-by-period interaction P value is 0.511 and is not included in the Holm adjustment.

Supplementary Table S9. Period-specific geometric mean ratios (GMRs) of plasma secreted protein acidic and rich in cysteine (SPARC) concentrations according to shunt-dependent hydrocephalus (SDHC) status in patients with bacterial infections, stratified by infection sites.

Infection site	Sampling period	No SDHC/SDHC, n	Unadjusted GMR (95% CI)	Holm-adjusted P value	Interaction P value
CNS infections					0.605
	Days 1–3	4/6	1.42 (0.64 to 3.18)	1.000	
	Days 4–6	4/6	1.00 (0.45 to 2.24)	1.000	
	Days 7–9	4/6	1.12 (0.50 to 2.51)	1.000	
	Days 10–12	4/6	1.69 (0.76 to 3.79)	0.748	
Non-CNS infections					0.846
	Days 1–3	13/6	1.32 (0.64 to 2.73)	1.000	
	Days 4–6	13/6	1.63 (0.79 to 3.37)	0.738	
	Days 7–9	13/6	1.15 (0.55 to 2.38)	1.000	
	Days 10–12	13/6	1.33 (0.64 to 2.75)	1.000	

Plasma SPARC concentrations were log-transformed before mixed-effects modeling. Each infection-site stratum is analyzed using a separate unadjusted mixed-effects model with a patient-specific random intercept. n denotes the number of patients with available measurements at each sampling period, presented as No SDHC/SDHC. GMRs are calculated as SDHC/No SDHC; values below 1 indicate lower model-estimated plasma SPARC concentrations in the SDHC group. The 95% confidence intervals (CIs) are pointwise and are not adjusted for multiplicity. P values are adjusted using the Holm method across the four period-specific contrasts within each infection-site stratum. Interaction P values refer to the SDHC-by-period interaction within each stratum and are not included in the Holm adjustment. Central nervous system (CNS) infections include meningitis (n = 9) and brain abscess (n = 1); non-CNS infections include pneumonia (n = 17), puncture-site infection (n = 1), and thigh abscess (n = 1).

Supplementary Table S10. Post hoc multivariable logistic regression analysis of the association between plasma secreted protein acidic and rich in cysteine (SPARC) concentrations at days 4–6 and shunt-dependent hydrocephalus (SDHC): sensitivity analysis including patients previously excluded due to bacterial infections, with additional adjustment for bacterial infection.

Variable	aOR (95% CI)	P value
Acute hydrocephalus	3.14 (1.57 to 6.29)	0.001
Admission WFNS grade V	2.36 (1.08 to 5.18)	0.032
Modified Fisher grade 4	1.75 (0.85 to 3.61)	0.131
Age, per 10-year increase	1.35 (1.05 to 1.73)	0.021
Bacterial infection	1.22 (0.49 to 3.03)	0.666
Plasma SPARC concentration at days 4–6, per 10 ng/mL increase	0.94 (0.88 to 1.00)	0.038

The model includes 213 patients (No SDHC, n = 148; SDHC, n = 65; and bacterial infection, n = 29). Adjusted odds ratios (aORs) for age and plasma SPARC concentrations are expressed per 10-year and 10 ng/mL increase, respectively. Reference categories are no acute hydrocephalus, admission World Federation of Neurological Surgeons (WFNS) grades I–IV, modified Fisher grades 1–3, and no bacterial infection. Bacterial infection denotes infection recorded during hospitalization; it does not indicate infection status at days 4–6. The upper 95% confidence limit for the SPARC aOR is 0.996 when expressed to three decimal places. CI, confidence interval.
